# The coherent organization of mental life depends on mechanisms for context-sensitive gain-control that are impaired in schizophrenia

**DOI:** 10.3389/fpsyg.2013.00307

**Published:** 2013-05-29

**Authors:** William A. Phillips, Steven M. Silverstein

**Affiliations:** ^1^Psychology, School of Natural Sciences, University of StirlingStirling, UK; ^2^Theoretical Neuroscience, Frankfurt Institute of Advanced StudiesFrankfurt, Germany; ^3^Rutgers University School of Biomedical and Health Sciences, University of Medicine and Dentistry of New JerseyPiscataway, NJ, USA

**Keywords:** cognitive coordination, context-sensitivity, gain-control, perceptual grouping, coherence, vision, schizophrenia, cortical computation

## Abstract

There is rapidly growing evidence that schizophrenia involves changes in context-sensitive gain-control and probabilistic inference. In addition to the well-known cognitive disorganization to which these changes lead, basic aspects of vision are also impaired, as discussed by other papers on this Frontiers Research Topic. The aim of this paper is to contribute to our understanding of such findings by examining five central hypotheses. First, context-sensitive gain-control is fundamental to brain function and mental life. Second, it occurs in many different regions of the cerebral cortex of many different mammalian species. Third, it has several computational functions, each with wide generality. Fourth, it is implemented by several neural mechanisms at cellular and circuit levels. Fifth, impairments of context-sensitive gain-control produce many of the well-known symptoms of schizophrenia and change basic processes of visual perception. These hypotheses suggest why disorders of vision in schizophrenia may provide insights into the nature and mechanisms of impaired reality testing and thought disorder in psychosis. They may also cast light on normal mental function and its neural bases. Limitations of these hypotheses, and ways in which they need further testing and development, are outlined.

## Introduction

Schizophrenia is well-known to be associated with disorganized and incoherent patterns of thought and behavior, but it is also associated with perceptual impairments that are less well-known because they are less obvious to casual observation. Substantial perceptual impairments have been rigorously demonstrated using many different experimental paradigms, however, and may constitute a core component of the illness. Furthermore, as so much is known at both psychological and neurobiological levels about the perceptual processes involved it may be easier to gain a deep understanding of those impairments than it is of the more obvious higher-level symptoms. If, as we argue here, perceptual and higher cognitive impairments arise from the same underlying pathophysiology, then this will show how perceptual impairments can provide a window on the disorder in general. The pathophysiology that we hypothesize to underlie many aspects of schizophrenia is centered on context-sensitive gain-control. Therefore, before reviewing evidence implicating it in schizophrenia, we provide an in-depth review of the arguments and evidence for its central role in normal mental life. Furthermore, we claim that schizophrenia research casts new light on context-sensitive gain-control, and assessment of that claim requires adequate knowledge of current views concerning its functions and mechanisms.

Put most simply, the perspective that we have been developing since the mid 1990s proposes that the coherent organization of mental life depends upon coordinating neural interactions of context-sensitive gain-control that amplify relevant signals and suppress irrelevant signals. Thus, disorganization and incoherence occur when those coordinating interactions are impaired, as in schizophrenia. Several other researchers in basic neuroscience have also argued that gain modulation, or gain-control, is a major principle underlying brain function (e.g., Salinas and Sejnowski, 2001; Chance et al., 2002). Gain-control changes the rate at which a neuron's output increases with the strength of the driving inputs to which it is selectively tuned. This suggests that neurons have two classes of input: one specifying selectivity and the other controlling gain. There is evidence that neuronal selectivity is specified by synaptic inputs that are few but strong whereas gain-control depends on many inputs that are individually weak (Lee and Sherman, 2010; Phillips et al., 2010). Gain-controlling inputs include those from the classical neuromodulators that have long been associated with the psychoses. Our theory also emphasizes gain-controlling interactions within and between glutamatergic and GABAergic neurons, however, because it is only they that convey the detailed cognitive content whose coherence is compromised in schizophrenia. It is not the inputs from sub-cortical neuromodulatory systems that convey that content, even though they do have modulatory effects on the intra-cortical interactions that do. Furthermore, there is ample evidence that impairments of the glutamatergic and GABAergic systems are central to schizophrenia.

Previous papers have distinguished gain-control from dynamic Gestalt grouping or “integration” (e.g., Butler et al., 2008), but here we use the phrase context-sensitive gain-control to cover both, because grouping can be seen as a form of context-sensitive gain-control on a fast time-scale that may use essentially the same mechanisms as other coordinating interactions (Phillips et al., 2010). The close relations between grouping and other forms of gain-control are clear in the similar dependence of contour integration and flanker facilitation on collinearity. Furthermore, there is clear evidence for deficits in both contour integration (Silverstein et al., 2009) and flanker facilitation (Must et al., 2004) in schizophrenia.

As context-sensitivity is central to our hypotheses we must also make clear what we mean by “context.” Some influential researchers identify “context” with information about time and place, or with information in working memory (WM) (e.g., Cohen and Servan-Schreiber, 1992; Gonzalez-Burgos and Lewis, 2008; Lewis, 2012). From our perspective those conceptions are far too narrow. Instead, we define context as any information that is used to modulate the strength with which a pyramidal cell, or local cortical circuit, transmits the information to which it is selectively sensitive. Thus, inputs arising from concurrent sensory input (e.g., Schwartz et al., 2007), as well as those arising from many other ongoing activities, can also control gain.

We do not provide a comprehensive review of the vast amount of empirical and theoretical research relevant to all the issues discussed in this paper. Our goal is to cite representative examples of the relevant findings. The perspective taken here overlaps in various ways and to various extents with several other theories. We do not claim priority for any of its essential components. One previous theory that we should relate it to, however, is our own. The central hypotheses examined are similar to those that we proposed ten years ago (Phillips and Silverstein, 2003), except that we now emphasize a greater variety of possible functions and mechanisms. Then we emphasized contextual disambiguation, now we emphasize various other possible functions. Then we emphasized NMDA receptors (NMDARs) for glutamate (the main excitatory neurotransmitter in cortex) as the main mechanism for gain-control. Now we emphasize several other possibilities also, including various classes of inhibitory interneuron and various intracellular mechanisms.

Thus, motivated by the above considerations, we examine five central hypotheses in this paper. First, context-sensitive gain-control is fundamental to brain function and mental life. Second, it occurs in many different regions of the cerebral cortex of many different mammalian species. Third, it has several computational functions, each with wide generality. Fourth, it is implemented by various mechanisms at cellular and circuit levels. Fifth, impairments of context-sensitive gain-control produce many of the well-known symptoms of schizophrenia and change basic processes of visual perception. To the extent that schizophrenia arises from widespread impairments of context-sensitive gain-control, insights gained from studying it can also inform our basic understanding of brain function and mental life in general.

These hypotheses are guided by various theoretical attitudes and working assumptions. First, our perspective is resolutely multi-disciplinary. Second, we assume that there are two-way causal interactions between macroscopic events at a psychological level and microscopic events at a cellular level. Third, we assume that impairments at synaptic and local circuit levels may be subtle, such as those related to differences between subtypes of cortical neuron or synaptic receptor. For example, there are various subtypes of NMDAR with the 2A and 2B subtypes being the most common in cerebral cortex. Small parametric differences in their biophysical properties suggest that the 2A subtype is better suited to operate on signals with high temporal precision. In addition, there is evidence that adolescence is associated with dramatic changes in NMDAR distribution (Wang and Gao, 2009), including a switch from the 2B to the 2A subtype (Liu et al., 2004). This suggests that disorders with an adolescent onset, as is the case with psychosis in schizophrenia, might be related to such subtle differences.

The remainder of this paper is organized into three sections. Section Context-Sensitive Gain-Control Plays a Central Role in Brain Function and Mental Life outlines our theory of context-sensitive gain-control, and reviews evidence concerning its role in normal cognition and brain function. Central aims of this section are to clarify our understanding of both its functions and its mechanisms. Section The Functions and Mechanisms of Context-Sensitive Gain-Control are Impaired in Schizophrenia reviews evidence that both the functions and the mechanisms are impaired in schizophrenia. Section Difficulties for the Hypotheses Proposed and Major Aspects to be Further Developed outlines some difficulties faced by the theory, and suggests ways in which it can be further tested and developed.

## Context-sensitive gain-control plays a central role in brain function and mental life

Contextual inputs to a local neural processor that arise from other ongoing activity in the system are in effect implicit predictions about the current state of the activity of that local processor. Mental life as a whole would be fully coherent if all local activities were as predicted by other ongoing activity. Though that state is an unrealistic ideal, we do depend on our ability to make sufficiently accurate inferences about distal things from proximal signals, about our own mental activities and intentions, and about the likely consequences of possible actions.

The centrality of unconscious inference to perception was emphasized by Helmholtz more than a 100 years ago. This perspective has now been extended into several prominent theories of cognition and brain function that are often referred to collectively as the “Bayesian brain” (e.g., Feldman, 2001; Purves et al., 2001; Körding and Wolpert, 2004; Yuille and Kersten, 2006; Friston and Stephan, 2007; Friston, 2010; Brown and Friston, 2012). The central tenet of these theories is that interpretation of sensory input depends upon the beliefs about the world that have been acquired from prior experience. Many psychophysical, neurobiological, and computational studies support this perspective (Clark, 2013). These theories usually imply a central role for context-sensitive gain-control, and they provide the basis for several recent and influential theories of schizophrenia (e.g., Friston, 2005; Corlett et al., 2007, 2010; Fletcher and Frith, 2009; Synofzik et al., 2010; Seth et al., 2011).

Our theory agrees with these main-stream theories in emphasizing probabilistic inference, but it differs in important ways. First, instead of relying primarily on “Bayes theorem,” we build on the foundations laid by the American statistical physicist Jaynes (2003). The many riches in Jaynes's analysis have been ignored by most neuroscientists and psychologists, but one notable exception is the neurophysiologist Fiorillo (2012) who sees the implications of Jaynes's logic as requiring radical changes in the current consensus concerning the “Bayesian brain.” We agree. The relevance of Jaynes's work to our perspective has therefore been examined in depth elsewhere (Phillips, 2012). Second, we assume that the requirements for optimal inference can rarely be fully met. For example, between what options must higher cortical regions choose when perceiving things? What prior data are relevant? On what should likelihoods be conditioned? These are not questions to which we expect optimal answers, even though, given answers to them, the principles of Jaynes's logic specify the optimal way to draw inferences from them. Third, context operates via the likelihoods, not via the prior probability, as shown formally by Kay and Phillips (2010). Fourth, we are not committed to the view that prediction error is the common currency of feed-forward signals between cortical regions as proposed by Rao and Ballard (1999). Instead, we assume that what is fed-forward from any level to the next is information about the current interpretations or predictions as inferred at that level. Fifth, inferences depend on contextual predictions, and hierarchical Bayesian theories emphasize feed-back signals from higher regions as the source of the predictions. We also emphasize predictions from lateral contextual interactions both within and between cortical regions, however, and this applies to all levels of the system as clearly demonstrated by the ubiquitous distribution of the mechanisms by which contextual interactions are implemented. All this is fully compatible with amplification of attended signals (Spratling, 2008; Spratling et al., 2009), and also with amplification of feed-forward signals that contradict strong predictions.

Our previous claims concerning the role of context-sensitive gain-control in cognitive coordination (Phillips and Silverstein, 2003) have been rigorously formalized using information theoretic concepts (e.g., Kay et al., 1998; Kay and Phillips, 2010), and are founded on cognitive, neurobiological, and clinical evidence, including that from visual psychophysics. The ubiquity of local ambiguity in visual perception and its resolution by context is well-established by many reviews of neurobiological and psychophysical studies (e.g., Phillips and Singer, 1997; Phillips and Silverstein, 2003; Butler et al., 2008; von der Malsburg et al., 2010). This applies to the early stages of visual processing, and also to the higher levels of perceptual interpretation, such as in the dependence of object recognition on scene context (Bar, 2004). Taking these well-established findings as a given, the perspective outlined here formalizes conceptions of context-sensitive gain-control rigorously in terms of computations that neural systems can perform, and relates them to detailed neurobiological mechanisms at both intracellular and local-circuit levels. These computations are described in formal terms to show that they can in principle deliver the capabilities claimed for them, and that they have sufficient generality to underlie many different domains of cognition.

Several prominent theorists have previously argued that gain modulation is a major computational principle underlying brain function (e.g., Salinas and Their, 2000; Salinas and Sejnowski, 2001; Chance et al., 2002), and some of its many computational functions will be listed in sub-section Context-Sensitive Gain-Control has Several Computational Functions. Within computational neuroscience gain modulation, or gain-control, is usually defined as a non-linear change in the response amplitude of a neuron that does not change its receptive field (RF) selectivity, i.e., its tuning function. Mathematically this has general utility because a population of responses to any RF variable, *x*, modulated by any context, *y* provides a basis set from which any function of *x* and *y* can be computed, and in many relevant cases it can be computed simply as a linear weighted sum (Salinas and Their, 2000; Salinas and Sejnowski, 2001). Salinas and Their (2000) note that to some researchers it can seem difficult to draw the line between selectivity and modulation, however, and that would greatly weaken any theory using it as a fundamental distinction. Fortunately, this crucial distinction is clear to others, such as Lamme (2004) whose extensive electrophysiological findings on contextual modulation led him to the conclusion that it bears no relation to the neuron's RF properties and is mediated by mechanisms far removed from those that shape and tune the local RF. Furthermore, the distinction can be rigorously formulated using information theoretic concepts (Smyth et al., 1996). Primary driving RF input is that determining the variables and values to which the neuron is selectively tuned, and about which it thus transmits information. Gain-control changes the rate at which the neuron's output increases with the strength of the driving inputs to which it is selectively tuned, but without fundamentally changing that selectivity. In short, selective driving inputs are both necessary and sufficient to produce an output signal; contextual inputs are neither necessary nor sufficient.

### Neurobiological evidence that context-sensitive gain-control occurs in many different cortical regions

There is ample evidence that context-sensitive gain-control occurs within the mammalian brain. Its wide distribution throughout the cortex is shown by evidence from single units, multiple units, local-field potentials, intra-cortical potentials, and macroscopic neuroimaging [see reviews by Phillips and Singer (1997); Phillips and Silverstein (2003); Lamme (2004); Schwartz et al. (2007); Salinas (2009); Lee and Sherman (2010); Feldman and Friston (2010); von der Malsburg et al. (2010)]. Studies using recent and revolutionary optogenetic techniques to control the activity of cortical cells also show that context-sensitive gain-control occurs in various cortical regions. They also provide clear evidence on the mechanisms by which gain-control is achieved, and so are reviewed in sub-section There are Various Local-Circuit and Cellular Mechanisms for Context-Sensitive Gain-Control where possible mechanisms are discussed. In addition to all the physiological evidence, common anatomical features of the canonical cortical circuit also suggest that gain-control is a general principle of cortical computation (Douglas and Martin, 2007, 2008).

Interpretation of these electrophysiological and anatomical findings has been strengthened by many computational studies of the role of context-sensitivity and gain-control in perceptual and higher cognitive functions. Examples include studies by Huang and Grossberg (2010) in learning and visual search, and many others reviewed by Schwartz et al. (2007) in relation to the perception of orientation. Context-sensitive gain-control is central to the computational model by which Schwartz et al. (2009) account for dynamic Gestalt grouping, the effects of context, and their dependence on natural scene statistics, all of which have been observed in visual cortex. Their model uses normalization in the form of divisive gain-control, and they argue that it is relevant to various levels of the visual system. Furthermore, contextual disambiguation and the dynamic grouping of coherently related elements may be of even greater importance to higher cognitive functions, such as language. Our working assumption is therefore that, as argued by Phillips and Singer (1997), context-sensitive gain-control provides a common foundation for cortical computation in general.

Evidence for a form of gain modulation that combines retinal and gaze signals multiplicatively was first observed in single-unit recordings in neurons of the parietal cortex of the macaque monkey, and computational studies showed that it could provide a basis for converting the position of stimuli relative to the retina into position relative to the head (Andersen et al., 1985). Since then several other coordinate transformations that could also be based on multiplicative gain modulation have been seen in other cortical areas (e.g., Galletti and Battaglini, 1989; Salinas and Sejnowski, 2001). This is widely assumed to be yet another form of context-sensitive gain-control, and that assumption will be reconsidered after our review of schizophrenia-related impairments because they cast new light on it.

### Context-sensitive gain-control has several computational functions

Context-sensitive gain-control has several computational functions; each with wide generality. Here we simply list some of the most well-known, making no attempt to review the substantial body of research available on each.

First, contextual disambiguation is one of its main functions. This could be achieved by multiplicatively increasing the gain on interpretations that are coherently related to the context and reducing the gain on those that are not. Examples of this include the enhancement of low-contrast edge detection by collinear flankers (Polat and Sagi, 1993), sensitivity of object recognition to scene context (Bar, 2004), word-sense disambiguation, and many other examples. Our broad conception of contextual disambiguation includes coordination of multiple distinct probabilistic decisions so that they form a coherent whole. An example of this at the level of object perception is the interpretation of ambiguous figures, such as the duck-rabbit figure or Necker cube. When perception switches between alternative interpretations it does so as a whole, implying that all the distinct decisions that this involves are coordinated by some form of context-sensitive gain-control that operates so as to maximize coherence over the whole figure that is being interpreted (Klemm et al., 2000).

Second, divisive normalization is another function of gain-control that has been described as a canonical computation. This has various uses from low levels of sensory processing to high levels of cognition such as value encoding (Carandini and Heeger, 2012). It includes surround suppression (Heeger, 1992; Simoncelli and Schwartz, 1999), invariant object recognition (Kouh and Poggio, 2008), the reduction of redundancy (Schwartz and Simoncelli, 2001), and various other ways of producing efficient codes. Recent neurophysiological findings show that input normalization by feedforward inhibition can expand the dynamic range of cortical activities by enabling populations of pyramidal cells to remain sensitive to weak inputs without saturating in response to stronger inputs (Pouille et al., 2009). Within the theory of Coherent Infomax, which provides the foundations on which our current hypotheses are built, the driving RF is equivalent to the driving summation field in normalization theory. The suppressive field in normalization theory is included within the contextual field (CF) that is an essential component of the Coherent Infomax theory. For an outline and peer-evaluation of that theory see Phillips and Singer (1997); for an outline and evaluation of its relevance to schizophrenia see Phillips and Silverstein (2003).

Third, context-sensitive gain-control can also play a role in dynamic Gestalt grouping. Grouping, sometimes referred to as “integration,” can be treated as a separate class of functions quite distinct from gain-control (e.g., Butler et al., 2008), but here we include it within a broad conception of gain-control for the reasons noted in section Introduction. Lamme (2004), who has studied contextual modulation extensively, argues that perceptual grouping is one of its main functions. Furthermore, there is much evidence that gain-control on a fast time-scale so as to synchronize coherent subsets could provide the basis for many cognitive functions including Gestalt figural organization (von der Malsburg et al., 2010). Finally, as we will show below, dynamic Gestalt grouping depends upon some of the same mechanisms as other forms of context-sensitive gain-control, such as fast-spiking inhibitory interneurons.

Fourth, context-sensitivity contributes to object and face recognition because the probability of seeing any given object or face depends so strongly upon the context (Bar, 2004). It may also contribute to the invariance of object recognition because normalization can be used to compute outputs that are insensitive to irrelevant stimulus dimensions (Salinas, 2009).

Fifth, selective attention also requires gain-control because it enhances the selected signals and suppresses the irrelevant signals. We therefore assume that the context that controls gain includes attention, which is in harmony with both the biased-competition theory (Desimone and Duncan, 1995), and its recent re-interpretation as a form of divisive normalization (Reynolds and Heeger, 2009).

Sixth, context-sensitive gain-control can produce efficient codes by using predictions to suppress the feed-forward transmission of signals that are highly probable, and thus not informative. Predictions are often assumed to be computed using hierarchical Bayesian inference (e.g., Lee and Mumford, 2003), and that possibility has now been developed into several highly influential theories. It may seem that such predictive coding theories are in conflict with the biased-competition theory of selective attention because they imply the suppression of predicted data, rather than its enhancement. It has been shown computationally that predictive coding and biased-competition are in principle compatible, however. This was done by combining them in a single model in which prediction-error processing occurs within, rather than between, cortical regions. Selective attention can then modulate those signals, so as to enhance, rather than suppress, the selected interpretations (Spratling, 2008; Spratling et al., 2009). Recent developments of that model show that by combining both driving and modulating inputs to the local processors it can account in detail for many well-established neurophysiological and psychophysical phenomena, including surround suppression, contour integration, predictive coding, and selective attention (Spratling, submitted).

Seventh, a neural network model has shown that contextual modulation can be used to select one of a number of possible arbitrary mappings from sensory stimuli to motor actions by controlling gain, thus helping to explain how higher organisms can rapidly and flexibly adapt their actions to current conditions (Salinas, 2004). Though that model is concerned with the selection of motor commands, the same computations could apply equally well to the selection of inner percepts and thoughts as assumed by the closely related theory of Coherent Infomax. Though these two theories were developed independently, they use essentially the same mathematical function to specify how the gain of the response to driving inputs is modulated by the context. Both theories are strengthened by this convergence because each provides further grounds for supporting the other.

Finally, another possible function is coordinate transformation, which was one of the first uses of gain modulation for which there was both empirical and theoretical evidence. This has been widely assumed to be a paradigmatic example of gain-control in general (e.g., Salinas, 2009), but the validity of that assumption remains to be determined.

It may also be possible to relate these basic computational functions of context-sensitive gain-control to more subjective aspects of human conscious experience. One recent development suggesting how that might be done is a theory of interoceptive inference which offers a unified account of emotion, the sense of presence, and the sense of agency (Seth et al., 2011), all of which are impaired in schizophrenia (e.g., Hauser et al., 2011) and can be impaired by drugs that block (modulatory) activity at NMDARs (e.g., Moore et al., 2013). By analogy with predictive coding theories of visual perception, interoceptive inference is hypothesized to involve a hierarchy of top-down predictions that guide the interpretation of bottom-up interoceptive signals. The subjective sense of the reality of the self and of the external world, referred to as conscious “presence,” is hypothesized to depend on the successful suppression of interoceptive signals by precise top-down predictions (Seth et al., 2011). Similarly, the subjective sense of agency is hypothesized to arise from precise predictions of the sensory consequences of actions, as proposed by Fletcher and Frith (2009). The theory of (Seth et al., 2011) synthesizes much of the relevant phenomenology, neurobiology, and psychopathology, and the precision of prediction error signals plays a key role in their theory. This is optimized by using context to control the gain of prediction error units. Seth et al. emphasize the role of the classic neuromodulators in doing this, and dopamine in particular, but more locally-specific coordinating interactions must also play a role. Their theory is particularly relevant here because it depends on the modulation of precision by gain-control, and explicitly shows how impaired gain-control could produce positive symptoms of psychosis. It is important to note that although Seth et al. emphasize feed-forward transmission of prediction-errors, rather than of the inferences used to make the predictions, that is not essential to predictive inference as explained in our above discussion of predictive coding.

### There are various local-circuit and cellular mechanisms for context-sensitive gain-control

There are various mechanisms for controlling gain within the cortex (e.g., Salinas, 2009; Silver, 2010). There is no simple one-to-one mapping between these mechanisms and the various functions of gain-control because one mechanism may contribute to more than one function and one function may be performed by more than one mechanism. Different mechanisms are suited to different roles, however, because they collect contextual information from very different sources, operate on very different time-scales, and vary greatly in the distribution of their effects, with some exerting gain-control that is highly local while others have widely distributed effects.

The simplest way in which pyramidal cells could increase the gain of other pyramidal cells so as to amplify coherent activities is via direct connections between them. It is likely that such a mechanism is used because it is the fastest and most energy efficient. In addition, it requires the transmission of a great deal of information and about 75% of all cortical connections are between pyramidal cells (Braitenberg and Schuz, 1991). Furthermore, NMDARs, which provide the means by which such connections can control gain (Phillips and Silverstein, 2003), are highly expressed on pyramidal cells. Recurrent excitation mediated by NMDARs also contributes to sustained neuronal firing, which is a potential substrate for WM (Gonzalez-Burgos and Lewis, 2008). Finally, a crucial role for direct gain-controlling interactions between pyramidal cells is shown by Self et al. (2012) using the well-established phenomenon of figure-ground modulation. This was almost abolished when NMDARs were blocked in V1, whereas the purely feedforward component of pyramidal cell response was largely unaffected. Conversely, blocking AMPA receptors did not affect figure-ground modulation, but did greatly reduce the feedforward component. Direct NMDAR-mediated interactions between pyramidal cells are therefore likely to be a widely used mechanism for controlling gain so as to amplify coherently related activities.

Recurrent connection between pyramidal cells requires tight inhibitory control to prevent runaway excitation, however, and this is provided by inhibitory interneurons. Much is now known about their role in shaping cortical activity (Isaacson and Scanziani, 2010), and they play a central role in various gain-control mechanisms. This is easy to understand intuitively: as inhibitory mechanisms for suppressing activity must be present it is likely that evolution has used them to control gain. It has recently been shown that this is so by combining advanced transgenic and optogenetic techniques with classical recording methods. Using optogenetic techniques experimenters can control the activity of genetically specified subtypes of cortical neuron in specified cortical layers of awake behaving animals, and they can do so with millimeter and millisecond precision. Studies using these techniques show that different classes of inhibitory interneuron provide mechanisms for different forms of context-sensitive gain-control. One major class, referred to as PV interneurons, are those expressing parvalbumin (PV), a low-weight protein involved in various physiological processes, including neuronal signaling. They have been identified with chandelier and basket cells, which are fast-spiking local-circuit inhibitory interneurons with synapses on perisomatic parts of pyramidal cells. Optogenetic studies show that under natural conditions PV interneurons can either amplify or suppress the gain of pyramidal cell activity (Atallah et al., 2012). They show that PV interneurons control the gain of the response of layer 2/3 pyramidal cells in an essentially simple way. Optogenetically suppressing PV interneuron activity increased layer 2/3 pyramidal cell activity multiplicatively by a factor of 1.2 and added a constant amount. Optogenetically activating PV interneurons decreased pyramidal cell activity divisively by a factor of 1.4 and subtracted a constant amount (Atallah et al., 2012). Furthermore, small changes in PV interneuron-mediated inhibition can lead to robust changes in the gain of pyramidal cell response without having any major impact on the selectivity of their tuning (Atallah et al., 2012). This provides direct and independent support for theories proposing that cortical computation is founded on the ability to control gain without fundamentally changing selectivity (Phillips and Singer, 1997; Kay et al., 1998; Phillips and Silverstein, 2003; Kay and Phillips, 2010).

Given that PV interneurons control gain, we need to consider the source of their inputs. One likely possibility is that this includes input from layer six cells in the same cortical column. Other optogenetic studies have revealed that excitatory cells in layer six of visual cortex control the gain of visually evoked activity in pyramidal neurons in the higher layers of the same cortical column (Olsen et al., 2012). This establishes pyramidal cells in layer six as a major mediator of cortical gain-control, so a major task for the future is to discover more about their inputs.

In addition to the PV-expressing class of inhibitory interneuron there is another large class, including Martinotti cells, which express the neuropeptide somatostatin (SOM interneurons). They are not fast-spiking and have axonal arbors on the distal dendrites of pyramidal cells. They are widely distributed across mammalian cortex, including that of humans, and are involved in the regulation of various processes. Of particular relevance here is their role in visual gain-control. By selectively reducing SOM interneuron activity using optogenetic techniques, it has been shown that they contribute to surround suppression (Adesnik et al., 2012). Reducing their activity significantly reduced surround suppression of layer 2/3 neurons by between 10 and 30%. SOM interneurons are but one of several mechanisms for surround suppression, however, as it is also in part inherited from earlier stages of visual processing, and is also in part due to other types of inhibitory interneuron and circuit mechanisms (Adesnik et al., 2012). A plausible default assumption is that visual surround-suppression by divisive normalization is but one example of a general computational strategy for suppressing highly probable signals, thus making less probable signals more salient (Seriès et al., 2003; Carandini and Heeger, 2012).

At the intracellular level there are several mechanisms by which gain-control can be implemented. These include shunting inhibition, background noise induced by balanced excitatory and inhibitory input, nonlinear dendritic integration such as dendritically localized NMDAR-mediated spikes, and short-term depression (STD) which can provide a mechanism for multiplicative gain-control if the contextual inputs are received on synapses distant from the cell body (Silver, 2010). Two mechanisms may be of particular relevance to the general computational issues considered here, as well as to schizophrenia (see below). One involves PV interneurons because they have the ability to amplify or suppress pyramidal cell activity. They also modulate temporal precision and generate synchronized gamma rhythms. They do this by controlling the “window-of-opportunity” within which pyramidal cells can generate spikes given their driving inputs (Gonzalez-Burgos and Lewis, 2008; Phillips et al., 2010). Computational modeling shows that by synchronizing the local activity of PV interneurons to a greater or lesser degree this window can be opened more or less. This is because PV neurons exert a powerful veto on spiking, so synchronizing their bursts also synchronizes the periods between bursts. This synchronized disinhibition therefore provides a “window of opportunity” for spiking that could provide a means by which contextual inputs, such as those from selective attention, could control the gain of pyramidal cell responses to their driving inputs (Tiesinga et al., 2008). It is known that in rodent primary somatosensory cortex their excitatory inputs are on distal dendrites, and come from both thalamic and intracortical sources, whereas their inhibitory inputs are somatic and perisomatic (Kameda et al., 2012), but we now need to know far more about those sources.

Another mechanism that may be of particular relevance is modulation of proximally driven activity by distal nonlinear dendritic currents, because that can either increase or decrease response gain at the soma (Silver, 2010). The possibility that distal dendritic tuft inputs might modulate response gain to inputs at the soma and basal dendrites was explored computationally by Körding and König (2000). They showed that this enables the learning and processing of information that is relevant to the context. Lee and Sherman (2010) distinguished two classes of glutamatergic pathways in the auditory cortex, termed “drivers” and “modulators.” Driving inputs are the information-bearing pathways, while modulators regulate transmission of the driving information. Driving inputs are received by proximal dendrites, whereas modulatory inputs are received by distal dendrites. Lee and Sherman (2010) also note that these two glutamatergic pathways are fundamentally different in other ways. Driving inputs are received from thick axons at ionotropic synapses, and produce large EPSPs via depressing synapses and dense synaptic arbors. Modulatory inputs are received from thin axons at ionotropic and metabotropic synapses, and they produce small EPSPs via facilitating synapses and sparse synaptic arbors. All these differences are in agreement with the distinction between driving inputs and context-sensitive gain-control on which our hypotheses here are based. Lee and Sherman (2010) argue that their distinction between drivers and modulators clarifies the function of the many parallel and descending pathways in the auditory and other sensory pathways. We agree, and argue for the potential relevance of such a distinction to cortical processing in general. Further support for the view that some contextual influences operate via thin distal dendrites is that the cortico-cortical projections that are likely to convey them terminate preferentially in superficial cortical layers and on the distal segments of apical dendrites of pyramidal cells, which are especially rich in NMDARs (Monaghan and Cotman, 1985; Rosier et al., 1993). We do not suggest that all contextual influences operate via distal dendrites, however. Inhibitory modulatory influences from PV cells are received on or proximal to the soma, so they do not operate via distal synapses. Furthermore, other mechanisms that are both modulatory and proximal may yet be discovered. A simple summary of the current evidence is that direct modulatory interactions between pyramidal cells seem to be predominantly distal, as does modulation by inhibitory SOM interneurons, whereas modulation by inhibitory PV interneurons is proximal to or on the soma.

Though much remains to be learned about the functions and mechanisms of context-sensitive gain-control, one important conclusion is already clear. It is not a single function with a single mechanism. It is a family of regulatory functions served by a variety of mechanisms, and with complex interactions between them; there is no need for evolution to produce only mechanisms that are easy for us to understand. The inhibitory interneuron activity that produces changes in pyramidal cell gain can itself be modulated by NMDAR-mediated input to the inhibitory interneurons. Thus pyramidal cells modulate each others' activities directly via NMDAR-mediated connections, and indirectly via their effects on the modulation produced by inhibitory interneuron activity. The different mechanisms nevertheless make different contributions as they are suited to different functions. It is going to be difficult to find out exactly which mechanisms do what because their capabilities depend on so many things (Silver, 2010). These include: (1) whether it is input or output gain that is modulated; (2) the morphological complexity of the cell whose activity is modulated; (3) whether the modulatory inputs are proximal to the soma or on distal apical dendrites; (4) whether the modulatory synapses are clustered or widely distributed; (5) whether the gain is to be increased multiplicatively or decreased divisively; (6) the time-scale over which gain is modulated; and (7) whether it operates on sustained high-frequency rate signals or on sparse and brief but temporally correlated population signals. Therefore, a major task for the cognitive neuroscience of the future is find out which of the various mechanisms for context-sensitive gain-control contribute to each of its various uses. This is a difficult task, but it may be greatly facilitated by studying disorders, such as schizophrenia, in which functions and mechanisms of context-sensitive gain-control are both impaired.

## The functions and mechanisms of context-sensitive gain-control are impaired in schizophrenia

Here we discuss visual and other impairments in schizophrenia in the light of the functions and mechanisms of context-sensitive gain-control reviewed above. As our focus is on impairments of basic capabilities common to many different cognitive domains and cortical regions, we are not constrained to consider only impairments that are specific to perception. We do need to ask whether or not they are specific to context-sensitive gain-control, however. The evidence suggests that schizophrenia-related impairments are rarely all-or-none, so our default working assumption is that the capabilities impaired are still operating to some extent, though less effectively.

### Impairments of visual perception in schizophrenia involve context-sensitive gain-control

Many studies show that impairments of visual perception in schizophrenia involve reduced context-sensitivity and gain-control. There is no need for a comprehensive review of all these impairments here because they will be the focus of other papers within this *Frontiers* Research Topic. Here it is sufficient to comment on recent assessments of this issue (Butler et al., 2008, 2012; Green et al., 2009), and to outline a few further findings.

Butler et al. (2008) divide the visual functions that are impaired in schizophrenic disorders into two groups, “gain control” and “integration.” They define gain control as processes optimizing response to stimuli within a particular surrounding context. One form of this is that in which the neurons' dynamic range is modulated so as to increase responses to differences between adjacent and successive stimuli, as seen, for example, in “pop-out” and “surround suppression” paradigms. Divisive gain normalization is the appropriate form of gain-control in that case, and center-surround suppression has been shown to be reduced in schizophrenia (Dakin et al., 2005; Yoon et al., 2009). Another form of gain control (Butler et al., 2008) is the amplification of driving inputs that are present but weak such as those produced by near-threshold stimuli, as shown, for example, by facilitation of the detection of a low-contrast edge by collinear flankers. This form of gain-control can be studied in various psychophysical and electrophysiological paradigms that measure contrast-sensitivity under conditions designed to reveal the operation of either the magnocellular or parvocellular visual pathway, and with either transient, moving, or steady-state stimulation. In general these paradigms include any in which the preceding, concurrent, or following context amplifies signals coherently related to that context. Multiplicative gain amplification is appropriate for this form of gain-control. It is clearly impaired in schizophrenia but not in other forms of serious mental illness (Butler et al., 2005; Kéri et al., 2005a,b, 2009). Impairment may be greater in magnocellular than in parvocellular pathways (Butler et al., 2005, 2008).

Butler et al. (2008) define “integration” as the process linking the output of neurons into globally coherent subsets, where their individual activities are assumed to code for local attributes. This is therefore equivalent to what is here and elsewhere referred to as dynamic Gestalt grouping. There are many paradigms for studying such grouping, with contour integration being an example that is often used because it can be rigorously controlled. Since 1961, many of these paradigms have been used to study visual grouping in schizophrenia (Snyder, 1961; Snyder et al., 1961), with the general conclusion being that it is impaired, as reviewed by Silverstein and Keane (2011). Impaired grouping in schizophrenia has been demonstrated in studies of perceptual organization of static forms, fragmented forms, completion of occluded objects, illusory correlations, and coherent motion. This evidence includes psychophysical, electrophysiological, and brain imaging data (e.g., Spencer et al., 2003; Silverstein et al., 2009; Sehatpour et al., 2010; Chen, 2011).

It is well-established that face processing is impaired in schizophrenia (e.g., Uhlhaas et al., 2006a; Turetsky et al., 2007; Silverstein et al., 2010; Soria Bauser et al., 2012), including changes in the perception of emotion (e.g., McBain et al., 2010), which can contribute much to disordered interpersonal interactions. As perceptual deficits are not confined to higher levels of processing, deficits at lower levels may account for some of the face processing impairments (Turetsky et al., 2007; McBain et al., 2010; Silverstein et al., 2010). Schizophrenia patients need more visual information and use it atypically (Lee et al., 2011), with configural or holistic processing being particularly impaired (Shin et al., 2008; Joshua and Rossell, 2009). All these findings harmonize well with the hypotheses we propose. Impairments in face perception are also observed in body dysmorphic disorder, the only other psychiatric condition in which perceptual organization impairments have been observed (Feusner et al., 2007, 2010), and where half of the patient population also exhibits delusional psychotic symptoms (Phillips et al., 2006).

Schizophrenia-related deficits have been shown to be specific to context-sensitive gain-control in experiments that use conditions in which context is misleading. If performance deficits are specifically due to reduced effects of context then performance may be supra-normal when context is misleading. This was shown to be the case in a size perception task where surrounding figures provided a context that was helpful in some conditions and misleading in others (Silverstein et al., 1996; Uhlhaas et al., 2006b). Schizophrenia patients in those studies were neither helped by helpful context nor hindered by misleading context. Similar results were reported by Dakin et al. (2005) who found that schizophrenia patients had decreased center-surround antagonism in a contrast perception task. High-contrast surrounds reduced perceived contrast of the central target in control subjects but not for most of the patients, with the consequence that patient's judgments were then more veridical than normal. Finally, Tadin et al. (2006) found that schizophrenia patients had reduced surround suppression in a motion perception paradigm, including more veridical performance in conditions where context was misleading.

Figure-ground segregation using brief temporal cues is also severely impaired in many but not all schizophrenia patients (Hancock et al., 2008). This was demonstrated in a task based on figure-ground segregation by onset-asynchrony. Performance in this task is likely to be particularly sensitive to the function of magnocellular pathways because it is concerned with rapid attentional capture, at low spatial resolution, of overall stimulus organization. Most people can segregate figure from ground when the asynchrony of their onsets is about 24 ms, but 7 of 9 chronically disorganized schizophrenia patients required asynchronies of at least 50–100 ms. (Hancock et al., 2008). Furthermore, 7 of 63 undergraduate students also showed poor temporal resolution in this task, four of whom had schizotypy disorganization scores well into the clinical range, suggesting that this psychophysical paradigm may provide a useful endophentoype for the disorder.

Eight possible uses for context-sensitive gain-control were listed in sub-section Context-Sensitive Gain-Control has Several Computational Functions. So far we have cited evidence that four are impaired in schizophrenia. What of the other four, i.e., selective attention, modulation of precision in probabilistic inference, arbitrary input-output mappings, and coordinate transformation? All are relevant to vision, though none to vision alone. Selective attention is clearly one of the major impairments in schizophrenia, and is related to positive symptoms (e.g., Cornblatt et al., 1985). Imprecise signaling in probabilistic inference may also make a major contribution to positive symptoms (e.g., Fletcher and Frith, 2009; Seth et al., 2011), as discussed further below. The use of context to guide selection of one from a number of possible mappings is also likely to be impaired, though we know of no work explicitly relating that to the model of Salinas (2004).

Finally, although it is often emphasized as a paradigmatic example of gain modulation in general (Salinas and Their, 2000; Salinas, 2009), coordinate transformation seems the least likely to be impaired in schizophrenia. Schizophrenia patients show no signs of disordered gaze or reaching, or of inadequate coordinate transformation in any other domain. Schizophrenia patients do demonstrate heightened spatial frame illusions, and this may suggest abnormalities in visuo-motor functioning (Chen et al., 2011). Schizophrenia patients do not demonstrate the normal degree of attenuation of sensory feedback during self-initiated movements, and this has been proposed as a factor in the formation of delusions of control by external entities (Landgraf et al., 2012). Similarly, schizophrenia patients do show heightened susceptibility to the rubber hand illusion, suggesting a more dynamic and flexible representation of their body in space (Thakkar et al., 2011). None of these findings suggest primary impairments in coordinate transformation, however. Maybe there are none. The obvious prediction from our theory is that coordinate transformation will only be impaired to the extent that it depends upon the same neuronal mechanisms as the forms of context-sensitive gain-control that are impaired. As many forms of context-sensitive gain-control are impaired but coordinate transformation seems not to be, this raises the possibility that, instead of being a paradigmatic example of gain-control in general, it is somehow quite different. Further thought on this issue reveals that neither of the two classes of input on which coordinate transformation depends meet our long-used criteria for classifying an input as “context,” because, according to our definitions, contextual inputs are neither necessary nor sufficient, whereas in coordinate transformation both classes of input that it combines are necessary. Knowledge of stimulus position relative to the retina and of eye-position relative to the head are both necessary to compute stimulus position relative to the head, for example. Thus, coordinate transformations depend on multiplicative interactions that are inherently symmetric as both terms are necessary. Driving and contextual interactions are inherently asymmetric, with context having a secondary, dependent, status (Phillips, 2012). Thus, this seems to provide a clear case where studies of visual impairments in schizophrenia contribute to our understanding of context-sensitive gain-control in general, because they indicate that, contrary to previous assumptions, it needs to be distinguished from coordinate transformation.

Overall, we can conclude that the context-sensitive perceptual operations of divisive gain suppression, multiplicative gain amplification, dynamic Gestalt grouping, and face and object perception are all impaired in schizophrenic disorders, though to different extents in different cases and conditions. This evidence shows that such impairments can occur at multiple levels of visual processing, and it suggests that they probably also occur in other modalities. These impairments are not constant over time. Some have been demonstrated to be state-sensitive in that they are more pronounced when patients are acutely psychotic compared to when their symptoms are in remission (Uhlhaas et al., 2005; Silverstein and Keane, 2009; Keane et al., in press; Silverstein et al., submitted, this research topic). Moreover, some of these state-sensitive impairments also occur in healthy volunteers administered ketamine, an NMDA antagonist (Uhlhaas et al., 2007; Morgan et al., 2011), as expected given the major contribution of NMDARs to gain-control according to our theory.

### Neuronal mechanisms for context-sensitive gain-control are impaired in schizophrenia

The classical neuromodulators that have long been implicated in schizophrenia, such as dopamine and acetylcholine, provide an obvious form of gain-control. Their effects are slow and diffuse, however, whereas the cognitive interactions that are most obviously impaired in schizophrenia must have high temporo-spatial specificity because they convey detailed cognitive content. Gain-controlling interactions within and between the glutamatergic and GABAergic systems that convey that content must therefore also be involved, so our focus here is on the evidence that they do indeed play a central role in the pathophysiology of schizophrenia.

Some of the interactions that control gain are produced via direct NMDAR-mediated interactions between pyramidal cells, as reviewed in sub-section There are Various Local-Circuit and Cellular Mechanisms for Context-Sensitive Gain-Control. There is ample evidence that NMDAR-mediated signaling is impaired in schizophrenia as reviewed many times elsewhere (e.g., Phillips and Silverstein, 2003; Loh et al., 2007; Corlett et al., 2010; Kantrowitz and Javitt, 2010; Moghaddam and Javitt, 2012). Furthermore, a review of the evidence on genetic susceptibility and gene expression concluded that, although there are probably direct and indirect links to both dopaminergic and GABAergic signaling, glutamate transmission via NMDARs is especially implicated (Harrison and Weinberger, 2005). Conclusive evidence that NMDAR hypofunction can produce many schizophrenic symptoms comes from an autoimmune disease first reported in 2007. This is an anti-NMDAR encephalitis that progressively reduces the activity of NMDARs by capping and internalizing them (Hughes et al., 2010). Patients present with acute schizophrenia-like symptoms, including paranoia. They are often admitted to psychiatric institutions and later develop severe catatonia, catalepsy, and stereotyped movement disorders. This provides unequivocal evidence that NMDAR-hypofunction can produce symptoms of schizophrenia, as we have long supposed.

Though less dense than on pyramidal cells, there are also NMDARs on the inhibitory interneurons on which several of the other mechanisms for context-sensitive gain-control discussed above depend. There is plenty of evidence that interneuron activity is also impaired in schizophrenia. Multiple studies have reported alterations in markers of inhibitory GABAergic neuronal activity (e.g., Lewis et al., 2005; Gonzalez-Burgos et al., 2010; Lewis, 2012), including their association with reduced center-surround suppression in visual cortex (Yoon et al., 2010). This deficit appears to be particularly pronounced in the subset of GABAergic neurons that express the calcium-binding protein PV (Hashimoto et al., 2003). Thus, this provides another route by which NMDAR-hypofunction could contribute to some of the deficits in schizophrenia. For example, when transgenic mice are generated in which NMDARs are selectively deleted from cortical and hippocampal GABAergic PV interneurons this produces selective molecular, physiological, and behavioral changes similar to some in schizophrenia (Nakazawa et al., 2012). Behrens and Sejnowski (2009) review evidence suggesting how dysregulation of PV interneurons in the developing cortex could explain the late onset of schizophrenic symptoms as well as the differences between the effects of brief and prolonged exposure to NMDA antagonists (Jentsch and Roth, 1999). The division of PV interneurons into two major classes is based on the principal target of their axon terminals. The axon terminals of the basket cell class target the cell body of pyramidal neurons and their proximal dendrites. The other major class, chandelier cells, gives rise to terminals that exclusively target the axon initial segments of pyramidal cells. There is evidence that both classes are impaired in a way that is specific to schizophrenia (Lewis et al., 2005; Lewis, 2012). PV interneurons also play a major role in setting the levels of temporal precision. This suggests that their impairments may play a major role in the reduced temporal precision of figure-ground segregation in schizophrenia reported by Hancock et al. (2008). Further evidence for the role of PV interneurons and synchronized rhythms in the development of schizophrenia is provided by Lee et al. (2012) who report that, in a neurodevelopmental rat model of schizophrenia, adolescent cognitive training changed PV-labeling in mature prefrontal interneurons, normalized the synchrony of neural oscillations between the left and right hippocampi, and prevented adult cognitive impairment.

Impairments of inhibitory interneuron activity could thus have several cognitive consequences. Many researchers, such as Lewis (2012), focus on consequences for WM and executive functions of the dorsolateral prefrontal cortex. We agree that dysfunctions of PV interneurons have consequences for WM and executive function, but from our perspective that provides far too narrow a focus as argued above. There is no good evidence linking the many selective impairments of perception reviewed here and elsewhere to WM or executive impairments. Effects of PV GABAergic impairment could also include many other cognitive functions as a consequence of their pivotal role in temporally precise activities including the generation and timing of rhythmic activity in the gamma frequency range (Cobb et al., 1995; Pouille and Scanziani, 2001). It is well-established that a wide range of cognitive deficits are associated with NMDAR-hypofunction and changed gamma-band activity in schizophrenia (Dzirasa et al., 2009; Uhlhaas and Singer, 2010). Uhlhaas and Singer (2012) review further evidence showing that synchronization of high-frequency rhythms is essential for the dynamic coordination of activities that are impaired in schizophrenia. They also summarize evidence suggesting that impaired long-range dynamic coordination of activity across brain-regions may be central to both of these disorders. The effects of impaired NMDAR-mediated neurotransmission on pyramidal cells and PV interneurons are particularly implicated. For example, the correlation between reduced GABAergic tone and reduced surround suppression in schizophrenia (Yoon et al., 2010) is probably mediated by gamma frequency oscillations, as recent research indicates a strong relationship between these three phenomena in healthy humans (Edden et al., 2009). Uhlhaas and Singer (2012) note many similarities between schizophrenia and autistic spectrum disorders (ASD) with respect to changes in rhythmic synchronization. Most of the paradigms used to study vision in schizophrenia have also been used extensively to study autistic perception, but very few firm conclusions can be drawn from all that evidence (Simmons et al., 2009). The possibility that a greater emphasis on context-sensitive gain-control as attempted here may reveal more order in all the evidence concerning both functions and mechanisms in ASD remains to be explored.

In addition to PV interneurons, other classes of inhibitory interneuron also contribute to context-sensitive gain-control. Evidence that somatostatin expressing interneurons (SOM interneurons), such as Martinotti cells, play a major role in surround suppression (Adesnik et al., 2012) was reviewed in sub-section There are Various Local-Circuit and Cellular Mechanisms for Context-Sensitive Gain-Control. There is evidence for SOM interneuron impairment in schizophrenia (Morris et al., 2008), and surround suppression is one of the forms of context-sensitive gain-control shown to be impaired in schizophrenia. Therefore, that impairment may be due to impairments of SOM interneuron activity.

Overall, the neurobiological evidence suggests that schizophrenia involves impairments of: (1) NMDAR-mediated interactions between cortical pyramidal cells, (2) the activities of PV inhibitory interneurons, and (3) the activities of SOM inhibitory interneurons. The interneuron impairments could, at least in part, be due to reductions in their NMDA-mediated synaptic input. All three mechanisms play a major role in context-sensitive gain-control, as outlined in section Context-Sensitive Gain-Control Plays a Central Role in Brain Function and Mental Life. An important direction for future research is therefore to relate these physiological impairments to the various signs and symptoms of schizophrenia. As noted above, there is growing evidence for: (1) state-sensitivity of impairments in context-sensitive gain-control in schizophrenia (Silverstein et al., 1996; Uhlhaas et al., 2005; Silverstein and Keane, 2009; Keane et al., in press; Silverstein et al., submitted); (2) relationships between reduced contextual effects in perception and fragmentation in thinking (Uhlhaas et al., 2006b; Horton and Silverstein, 2011; Silverstein and Keane, 2011); and (3) relationships between abnormal GABAergic activity and context-sensitive gain-control in schizophrenia (Yoon et al., 2010). Symptoms are, by definition, state related, and many theories now relate positive and disorganized symptoms of psychosis to altered states of NMDARs and interneuron activity. However, the development of pharmacotherapy on the basis of these theories, though promising, has not yet clearly improved on clozapine, which has been available for 50 years (Barch, 2010; Moghaddam and Javitt, 2012). This may, in part, be due to the difficulty of specifying clinically optimal doses. It could also be related to the need to distinguish between subtypes of receptor and post-synaptic cell. For example, if impairments are due to reduced activity of only a particular NMDAR subtype on a particular class of post-synaptic cell, then that would not be overcome by a systemic enhancement of NMDAR activity in general. Therefore, we need a better understanding of the different functional roles and developmental trajectories of the different subtypes of NMDAR.

### The positive symptoms of schizophrenia are related to context-sensitive probabilistic inference and gain-control

Psychiatrists have often concluded that contextual regulation of ongoing processing is particularly relevant to the induction of thought disorder (e.g., Barrera et al., 2005). Over the last few years this possibility has been developed into rigorously formulated theories that focus on the use of context to guide processing toward inferences that are both coherently related to each other and well adapted to the current circumstances. These theories often assume a form of hierarchical Bayesian inference that adapts and learns by reducing prediction error, where the predictions arise from higher levels of processing (e.g., Friston, 2010). In addition, we emphasize that predictive inputs are also provided by lateral interactions within levels. Such theories have been used to explain hallucinations (e.g., Friston, 2005) and various forms of delusion (e.g., Hemsley and Garety, 1986; Garety et al., 2001; Corlett et al., 2007; Fletcher and Frith, 2009; Clark, 2013). In essence, to the extent that perception is under-constrained by prior experience of statistical regularities in the world, misperceptions and false attributions of meanings can result. These can produce a sense that the world is changing, giving rise to delusional explanations for these subjective changes. Delusions of agency are well-explained by these models on the assumption that they arise from reduced precision in the predictions of self-induced sensory signals (Fletcher and Frith, 2009; Stephan et al., 2009; Synofzik et al., 2010). In section Context-Sensitive Gain-Control Plays a Central Role in Brain Function and Mental Life we cited work showing how theories of this kind can explain the normal sense of conscious presence as arising from the correct prediction of interoceptive signals (Seth et al., 2011). That theory explains how disorders of both conscious presence and emotion could arise from reductions in the context-sensitivity and precision of probabilistic inference. Such theories can explain many of the psychotic symptoms that are seen in schizophrenia patients. Thus, they may provide important insights into the well-established symptoms of schizophrenia, and all depend upon context-sensitive gain-control. They imply a distinction between drivers and modulators because the predictions that are central to these accounts are thought to be modulatory and implemented by specialized synaptic interactions, such as those using NMDARs and inhibitory interneurons. Though we are not convinced by some aspects of the theories based on predictive coding (Phillips, 2013; Silverstein, in press), we agree with their emphasis upon the necessity of using probabilistic inference to interpret interoceptive inputs as well as those from the external world, and we emphasize the role of context-sensitive gain-control in doing that. Many of the positive symptoms of schizophrenia can thus be seen as arising from predictions that are pathologically imprecise because inadequate use is made of context to make them more precise. The use of contextual modulation can also enable the selection of perceptual interpretations or motor commands that have low probability overall, but high probability in special contexts. Thus, in addition to the symptoms noted above, weakened context-sensitivity could also lead to various other impairments of perception, thought, and action. Recent evidence in support of this is that reduced application of a convexity prior during perception of a hollow mask can lead to more veridical perception of such stimuli by schizophrenia patients. Furthermore, the extent of veridical perception by such patients was related to higher levels of hallucination and delusion, and to fewer days since last hospital discharge (Keane et al., in press). Moreover, this reduced sensitivity to the “hollow-mask illusion” has been shown, in dynamic causal modeling analyses of ERP and fMRI data, to be due to reduced top-down modulation of occipital lobe output in people with schizophrenia (Dima et al., 2009, 2010), as our theory predicts.

## Difficulties for the hypotheses proposed and major aspects to be further developed

Hypotheses as general and abstract as ours cannot be confirmed or refuted by a single definitive experiment. Nevertheless, they can be strengthened or weakened by further evidence. For example, if further studies reveal many perceptual deficits in schizophrenia that are neither primary nor secondary consequences of impaired context-sensitive gain-control then our hypothesis concerning the functional impairments in schizophrenia would need to be amended. It will therefore be of great interest to see whether other papers published as part of this *Frontiers* Research Topic reveal such deficits. If schizophrenia were shown to be due to impairments of mechanisms unrelated to context-sensitive gain-control then our hypothesis concerning the neuronal bases of schizophrenia could be rejected. Our hypotheses carry many implications concerning mechanisms that can be tested and developed by further work. Indeed, differences between our emphases now and those in Phillips and Silverstein (2003) show this clearly. Then we placed great emphasis on the role of NMDAR-mediated interactions between pyramidal cells as the mechanism for context-sensitive gain-control. Now we also place great emphasis on the role of PV interneurons because recent findings, such as those using optogenetic techniques, demonstrate that they are well-suited to perform context-sensitive gain-control (Atallah et al., 2012).

Most fundamentally, our hypotheses depend upon the distinction between context-sensitive gain-control and the driving signals that convey content. If that distinction were shown to be misleading or of no use then our perspective could be justifiably ignored. Though many arguments and findings have been offered in favor of such a distinction by ourselves and others, some researchers remain unconvinced, so we acknowledge that this fundamental distinction remains open to question. Furthermore, we assume that presentation of the distinction as dichotomous is merely a heuristic simplification, but we have not given examples of cases that are intermediate between the two poles of the distinction.

Theories founded on the notion of optimal Bayesian inference have been challenged in various ways (e.g., Jones and Love, 2011). For example, Bowers and Davis (2012) argue that such theories are difficult to test because *post-hoc* assumptions about priors or likelihoods can be used to explain almost anything. They also argue that human inference is often not optimal, and that the neurobiological evidence for such theories is weak. Clark (2013) also notes that, being founded on the narrow goal of reducing prediction error, these Bayesian theories present a bleak desert-landscape view of mental life. Though most commentators on his *Behavioural and Brain Sciences* target article support his enthusiasm for predictive processing, several raise other difficulties that our perspective may help reduce. First, one difficulty often raised concerns optimality, but we do not assume optimality. On the contrary, we argue that the conditions for optimality at the systems-level can be met only in exceptionally simple cases (Phillips, 2012). Second, the neurobiological evidence for our hypotheses is strong and rapidly becoming stronger as it is supported by the optogenetic evidence that is now being used to explore the mechanisms of context-sensitive gain-control. Third, the theory of Coherent Infomax that underlies the hypotheses proposed in this paper avoids the desert-landscape criticism by emphasizing the objective of maximizing coherent inference rather than that of reducing prediction error (Phillips et al., 1995; Kay and Phillips, 1997, 2010; Phillips, 2013). Finally, another difficulty facing any simple unifying theory is the need to explain the endless diversity of cognitive capabilities. Our perspective has to some extent met this need by showing that context-sensitive gain-control in visual size-perception varies greatly across people of different ages (Doherty et al., 2009), sex (Phillips et al., 2004), and culture (Doherty et al., 2008), but those studies are merely the first few steps into a largely unexplored territory.

Plenty of other difficulties and undeveloped possibilities remain. We cannot yet claim that all of the symptoms associated with schizophrenia are due to impairments of context-sensitive gain-control or their secondary consequences. Nor do we yet have fully adequate answers to questions concerning relations between schizophrenia-related impairments and the coordinate transformations that some see as a foremost function of gain-control. Is coordinate transformation impaired in schizophrenia or not? If not, why not? Is it because the form of gain-control involved in coordinate transformation is not context-sensitive in the way that the others are? Relations between classical neuromodulation and the more locally specific gain-control that we have emphasized also need to be further clarified. We expect them to be complex, and to operate in both directions. There is also much that needs to be clarified concerning the full range of schizophrenia-related deficits in visual perception. For example, it is well-established that these include changes in visual masking (Green et al., 2011). Such deficits may be related to the reduced temporal precision shown by Hancock et al. (2008) and to the impairments of PV inhibitory interneurons emphasize above, but we have not yet examined that possibility adequately.

Overall, our view of the difficulties and immaturities faced by our perspective is that they offer far more opportunity for healthy growth than they do for fatal decline. It will be of great interest to see whether developments over the coming years justify that optimism.

### Conflict of interest statement

The authors declare that the research was conducted in the absence of any commercial or financial relationships that could be construed as a potential conflict of interest.
